# The Adverse Drug Reactions From Patient Reports in Social Media Project: Protocol for an Evaluation Against a Gold Standard

**DOI:** 10.2196/11448

**Published:** 2019-05-07

**Authors:** Armelle Arnoux-Guenegou, Yannick Girardeau, Xiaoyi Chen, Myrtille Deldossi, Rim Aboukhamis, Carole Faviez, Badisse Dahamna, Pierre Karapetiantz, Sylvie Guillemin-Lanne, Agnès Lillo-Le Louët, Nathalie Texier, Anita Burgun, Sandrine Katsahian

**Affiliations:** 1 INSERM U1138 - Team 22 Information Sciences to Support Personalized Medicine Centre de Recherche des Cordeliers Paris France; 2 Département d'Informatique Médicale Hôpital Européen Georges-Pompidou Assistance Publique - Hôpitaux de Paris Paris France; 3 Innovative Projects - Text Mining Expert System Paris France; 4 Centre Régional de Pharmacovigilance Hôpital Européen Georges-Pompidou Assistance Publique - Hôpitaux de Paris Paris France; 5 Kappa Santé Paris France; 6 Service d'Informatique Biomédicale, D2IM Centre Hospitalier Universitaire de Rouen Rouen France; 7 INSERM U1138 - Team 22 Information Sciences to Support Personalized Medicine Paris Descartes University, Sorbonne Paris Cité Paris France; 8 Clinical Research Unit Hôpitaux Universitaires Paris Ouest Hôpital Européen Georges-Pompidou Assistance Publique - Hôpitaux de Paris Paris France; 9 INSERM CIC1418 Clinical Epidemiology Hôpital Européen Georges-Pompidou Paris France

**Keywords:** social media, drug-related side effects and adverse reactions, natural language processing, data mining, MedDRA, Racine Pharma

## Abstract

**Background:**

Social media is a potential source of information on postmarketing drug safety surveillance that still remains unexploited nowadays. Information technology solutions aiming at extracting adverse reactions (ADRs) from posts on health forums require a rigorous evaluation methodology if their results are to be used to make decisions. First, a gold standard, consisting of manual annotations of the ADR by human experts from the corpus extracted from social media, must be implemented and its quality must be assessed. Second, as for clinical research protocols, the sample size must rely on statistical arguments. Finally, the extraction methods must target the relation between the drug and the disease (which might be either treated or caused by the drug) rather than simple co-occurrences in the posts.

**Objective:**

We propose a standardized protocol for the evaluation of a software extracting ADRs from the messages on health forums. The study is conducted as part of the Adverse Drug Reactions from Patient Reports in Social Media project.

**Methods:**

Messages from French health forums were extracted. Entity recognition was based on *Racine Pharma* lexicon for drugs and Medical Dictionary for Regulatory Activities terminology for potential adverse events (AEs). Natural language processing–based techniques automated the ADR information extraction (relation between the drug and AE entities). The corpus of evaluation was a random sample of the messages containing drugs and/or AE concepts corresponding to recent pharmacovigilance alerts. A total of 2 persons experienced in medical terminology manually annotated the corpus, thus creating the gold standard, according to an annotator guideline. We will evaluate our tool against the gold standard with recall, precision, and f-measure. Interannotator agreement, reflecting gold standard quality, will be evaluated with hierarchical kappa. Granularities in the terminologies will be further explored.

**Results:**

Necessary and sufficient sample size was calculated to ensure statistical confidence in the assessed results. As we expected a global recall of 0.5, we needed at least 384 identified ADR concepts to obtain a 95% CI with a total width of 0.10 around 0.5. The automated ADR information extraction in the corpus for evaluation is already finished. The 2 annotators already completed the annotation process. The analysis of the performance of the ADR information extraction module as compared with gold standard is ongoing.

**Conclusions:**

This protocol is based on the standardized statistical methods from clinical research to create the corpus, thus ensuring the necessary statistical power of the assessed results. Such evaluation methodology is required to make the ADR information extraction software useful for postmarketing drug safety surveillance.

**International Registered Report Identifier (IRRID):**

RR1-10.2196/11448

## Introduction

### Background

The detection of new adverse drug reactions (ADRs) has been based on postmarketing surveillance by government agencies (national authorities) derived from spontaneous reporting by health care professionals and patients. The US Food and Drug Administration (FDA) and the European Medicines Agency collect ADR case reports through the FDA’s Adverse Event Reporting System (FAERS) [1-4] and the EudraVigilance system, respectively. These systems are useful tools for drug agencies, which mine these huge amounts of structured data to look for new safety concerns that might be related to a marketed product [5-8].

In the last 20 years, internet and social media have become an integral part of people’s daily life. Social media is now often used to communicate with other persons having the same health concerns and share information regarding their health conditions, feelings, medications, and many other aspects [9]. Social media is, therefore, a potential provider of information on ADRs. In 2005, the International Society of Drug Bulletins already recognized such a use: *Patient reporting systems should periodically sample and evaluate the scattered drug experiences patients report on the internet* [10]. This new source of knowledge captured the interest from health informatics, statistics, and public health researchers. Although in its infancy, related scientific literature increased in the last decade [4,11-13].

### Objectives

In this context, the French Ministry of Industry funded and launched the Adverse Drug Reactions from Patient Reports in Social Media (ADR-PRISM) project. The objective of ADR-PRISM was to make available the contents about ADR, informal and embedded in forums and discussions on the Web, to the actors involved in drug safety (drug companies, agencies, and pharmacovigilance experts). In the end, the tool developed in the ADR-PRISM project should generate hypotheses about new or poorly documented adverse events (AEs). To reach its goals, the ADR-PRISM consortium gathers a company developing text mining softwares (Expert System), a company specialized in pharmaco-epidemiology (Kappa Santé), 3 academic research groups providing expertise in medical informatics and statistics (National Institute of Health and Medical Research & Cordeliers Research Centre: umrs 1138 team 22 dedicated to Information Sciences to support Personalized Medicine and Laboratory in Medical Informatics and Knowledge Engineering in e-Health, and Biomedicine informatics, Service Catalogue and Index of French Language medical websites SIBM-CISMeF), 2 experts in pharmacovigilance (regional center of pharmacovigilance), as well as Vidal group that supplies the drug database used in most drug prescription systems in France.

From a natural language processing (NLP) perspective, we considered ADR as relationships between drug and AE concepts. On the basis of that, the NLP-based Skill Cartridge for pharmacovigilance developed by Expert System for ADR-PRISM includes a relation extraction module based on (named) entity recognition combined with rules and regular expressions. Before applying it to large collections of forum discussions, we designed a protocol to assess the performance of this ADR information extraction module. The objective of this study is to present this protocol.

## Methods

### Synopsis

With the objective of sharing a methodology that guarantees the confidence in the results in ADR-PRISM, we followed a way of reasoning inspired by the standards widely adopted in clinical research such as the Standards for Reporting Diagnostic Accuracy studies [14]. These items are displayed in Textbox 1.

**Synopsis of the protocol.**

Rationale

- Nowadays, patients extensively use social media. They report on the adverse events they feel because of their medications (further called adverse drug reaction [ADR]) on health forums. Promising studies exist on the extraction of ADR information from social media with natural language processing (NLP) or machine learning tools.
- The consortium Adverse Drug Reaction from Patient Reports in Social Media (ADR-PRISM) has been constituted to create a tool extracting the ADR information from social media. Teams specialized in text mining NLP and pharmacovigilance participate in the consortium.
- Before applying the ADR-PRISM’s tool on a larger scale (millions of posts) to draw conclusions on ADRs, our goal was to adapt the principles adopted in clinical research to assess the ADR-PRISM. For example, evaluation was done against a gold standard based on manual annotation of the posts.

Primary objective

- To estimate robustly the performance of the ADR information extraction tool against gold standard.

Primary expected results

- Precision, recall, and f-measure for ADR extraction.

Secondary objectives

- To verify the quality of the gold standard and to evaluate the impact of various conditions (eg, different granularities and sentence constraint of the tool) on the performance of the ADR information extraction tool.

Secondary expected results

- Kappa metrics for interannotator agreement; and precision, recall, and f-measure for ADR extraction in various conditions.

Database

- Posts from the Kappa Santé Detec’t database published between January 1, 2007, and October 28, 2016.

Eligibility criteria

- Posts from the Kappa Santé Detec’t database that contain at least one drug’s or molecule’s (active substance) name, and posts randomly selected from the rest of the Kappa Santé Detec’t database; a list of drugs and adverse events (AEs) of interest is established by drug safety experts; and ADR: any explicit and positive relationship between a drug and a potential AE where either the drug or the AE or both belong to the list.

Index test method

- ADR information extraction tool: this tool classifies each co-occurrence of drug and AE in a post as positive ADR or negative ADR or no ADR, and maps the drug and the AE expression to *Racine Pharma* and Medical Dictionary for Regulatory Activities (MedDRA), respectively.

Reference method

- Gold standard: manual annotations of the co-occurrence of drug and AE as positive ADR or negative ADR or no ADR including the mapping of the drug and the AE expression with a *Racine Pharma* and a MedDRA entity, respectively. Manual annotations will be provided by 2 annotators with experience in medical terminology.

Sample size

- A 95% CI, with a total width of 0.1, is used to determine the sample size.

Statistical analyses

- Recall, precision, and f-measure calculations for evaluation of the performances of the ADR extraction information tool and interannotator agreement for the evaluation of the gold standard with a Cohen kappa.

### Design and Ethics

#### Design

This project is based on retrospective data collected among threads of discussion accessible on social media (messages from French health forums). A total of 2 experts in pharmacovigilance helped delineating the project. The objective is twofold: (1) focus on certain pharmaceutical products of interest and identify the related ADRs and (2) detect the emergence of general potential problem in public health. On this basis, the extraction of ADR information is expected to (1) perform well on specific concepts for use case study and (2) be able to extract all potential concepts correctly for screening purpose.

**Figure 1:**
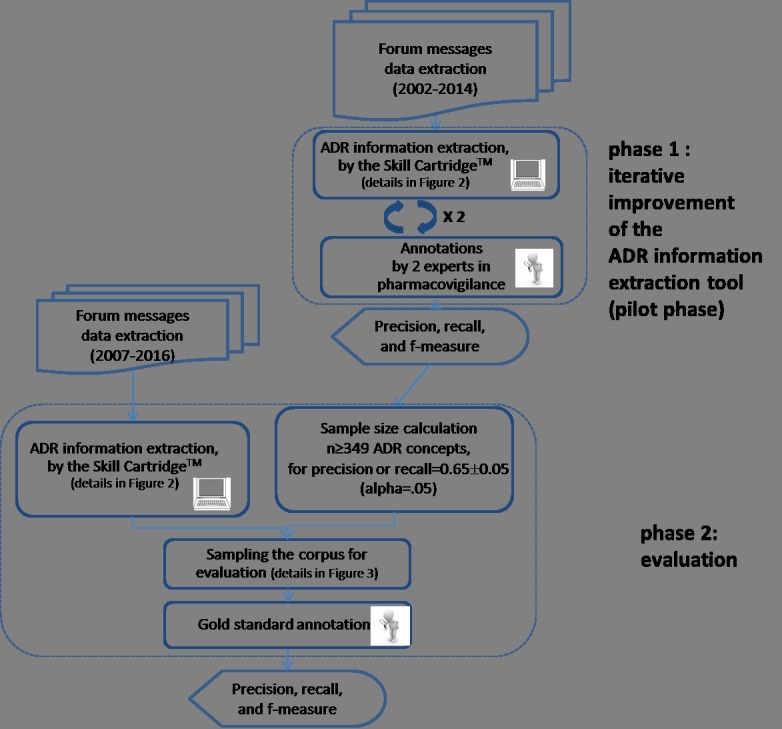
Study overview. ADR: adverse drug reaction.

To evaluate the ADR information extraction, we therefore build up the global process in 2 phases (Figure 1). In phase 1, we implemented an iterative process of validation and improvement of the Skill Cartridge for pharmacovigilance. The objectives were (1) to correct the most frequent errors and to train the ADR information extraction module and (2) to estimate the performance indicators and the time required for manual annotation for phase 2 sample size determination. Then, in phase 2, we will conduct a definite assessment of the performance of the ADR information extraction module.

In the rest of the paper, we use the term *AE* for medical events that are present in text for a potential ADR, whereas the term *ADR* is used when a relation between a drug and an AE is established.

#### Ethics

This research did not involve experiment on either humans or animals. Ethics and guarantee of data privacy constituted an integral and dedicated working group set up for the ADR-PRISM project. To comply with national regulations, we first registered data collection to the French Data Protection Agency (CNIL for Commission Nationale Informatique et Liberté), which is known as a *normal notification* in technical terminology, on December 23, 2015. We later submitted an authorization request on March 30, 2016, regarding data analysis and validation of the approach adopted about ethics and confidentiality to the same agency. ADR-PRISM’s consortium detailed approach is explained in the study by Bousquet et al [15].

The ADR-PRISM project was supported by an ethics advisory board, which was composed of scientists with different scientific expertise: drug safety, public health, and ethics. Their role was to give independent advice regarding ethical issues to the project consortium. The board approved the whole study design, including the protocol presented in the paper.

### Adverse Drug Reactions From Patient Reports in Social Media Adverse Drug Reaction Extraction

#### Resources

We used 3 lexicons to represent drug information and AEs. We codified AEs with the Medical Dictionary for Regulatory Activities (MedDRA) v15.1 [16] classification. *Racine Pharma*, maintained by the SIBM-CISMeF [17], provided an extensive source of drug names that covered all medications available on the French market, including brand names and active ingredients. *Racine Pharma* entries were mapped to the Anatomical Therapeutic Chemical (ATC) system [18], which was used as a classification system for drugs.

The corpus of messages was extracted from the Detec’t database [19], a database developed by Kappa Santé that collects messages from several French forums using a Web crawler. We limited ourselves to a list of 5 major health forums in French obtained using netscoring [9]. Message extraction was based on a named entity recognition module using a drug lexicon designed by Kappa Santé and a fuzzy matching algorithm. The lexicon was based on *Racine Pharma*, the ATC classification, and a list of medications extracted from the French National Health Insurance database.

### Drugs, Adverse Events, and Adverse Drug Reaction Extraction

**Figure 2:**
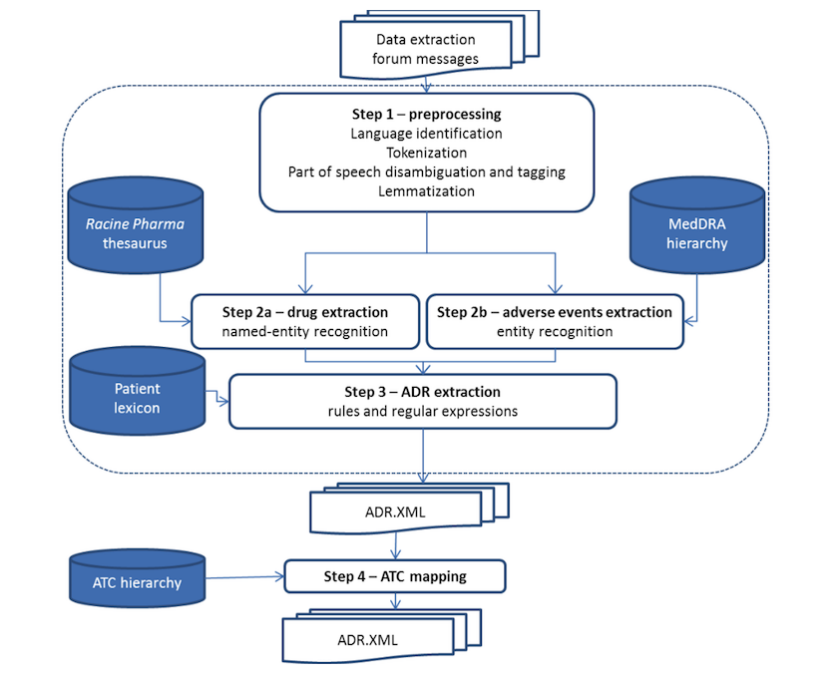
Adverse drug reaction pipeline. ADR: adverse drug reaction; ATC: Anatomical Therapeutic Chemical (in ATC classification); MedDRA: Medical Dictionary for Regulatory Activities.

ADR extraction was performed in 2 steps: first, named entity recognition modules were used to identify drug names and AEs in posts, and then, a relation extraction algorithm was applied to these entities (Figure 2).

#### Drugs and Adverse Events Extraction

Regarding drugs, Expert System has developed a named entity recognition module capable of identifying words or tokens (occurrences) listed in *Racine Pharma* and extracting their positions from forum posts.

We mapped the extracted expressions from *Racine Pharma* to the chemical substance level (5th level) of the ATC (last updated version: December 19, 2016) [18]. Considering that the same active ingredient could be found under different trade names, we pooled all mapped expressions within the same ATC chemical subgroup (4th level) to define the drug concepts.

Expert System has developed another module based on MedDRA to identify the AEs and extract their position in the posts. Fuzzy-matching and enrichment of thesaurus with colloquial terms enabled to take into account the characteristics of the posts on health forums.

#### Drug and Adverse Event Relationship Extraction (Skill Cartridge for Pharmacovigilance)

An NLP module has been developed by Expert System to capture the specific information regarding the relationship established between a drug and an AE by the post’s author. The module combined a set of rules and regular expressions, with a *Patient* lexicon constituted to ensure that the post’s author set out a situation of a person taking the drug and experiencing the symptom and excludes general information regarding a drug or an AE. This lexicon contains terms such as *I*, *me*, *my*, *cause*, *test*, *take*, *feeling*, *because of*, *provoke*, *intolerance*, and *allergies*. This NLP module also included negation detection.

The algorithm can be summarized as follows:

1. Text was split into sentences based on the punctuation mark.
2. For each sentence, (named) entity recognition modules extracted drugs and AEs.
3. For each pair of drug and AE co-occurring in a sentence, if the NLP module extracted specific information regarding a relation, then the co-occurrence was classified as follows:
  - Explicit and positive ADR (eg, *Abilify causes me such fears that I cannot concentrate to read, work, etc*...);
  - Explicit and negative ADR (eg, *I took some Doliprane and I didn’t feel any nausea);*
  - If no specific information was identified, the co-occurrence was classified as no ADR (eg, *Usually, Focalin’s adverse effects are loss of appetite, insomnia, and naso-pharyngitis*).

### Phase 1: Iterative Improvement of the Adverse Drug Reaction Information Extraction Module

Phase 1 consisted of an iterative process of validations and improvements of the tool. The review on the corpus was constituted by manual annotation of the messages by 2 experts in pharmacovigilance. The manual annotation process used a tool developed by Expert System for in-house testing purposes [20]. This tool did not integrate functionalities for blind manual annotation. No gold standard was established at this step. All along the process, both annotators could see the drugs, AEs, and ADRs extracted by the tool, and their task consisted of validating or invalidating these extractions and, if needed, complementing the annotations performed by the system. The annotators were asked to (1) check all drugs and all AEs in the post, irrespective of whether they have been involved in an ADR relationship, and (2) identify all drug-AE relationships in the same sentence. The output of phase 1 was a first estimation of precision, recall, and f-measure, assessing the capacity to extract information regarding drugs, AEs, and ADRs. More details about the corpus construction, evaluating concept selection, sample size determination, and the preliminary results of this phase were reported in the study by Chen et al [21].

### Phase 2: Evaluation

#### Corpus for Evaluation

##### Evaluation Dataset

**Figure 3:**
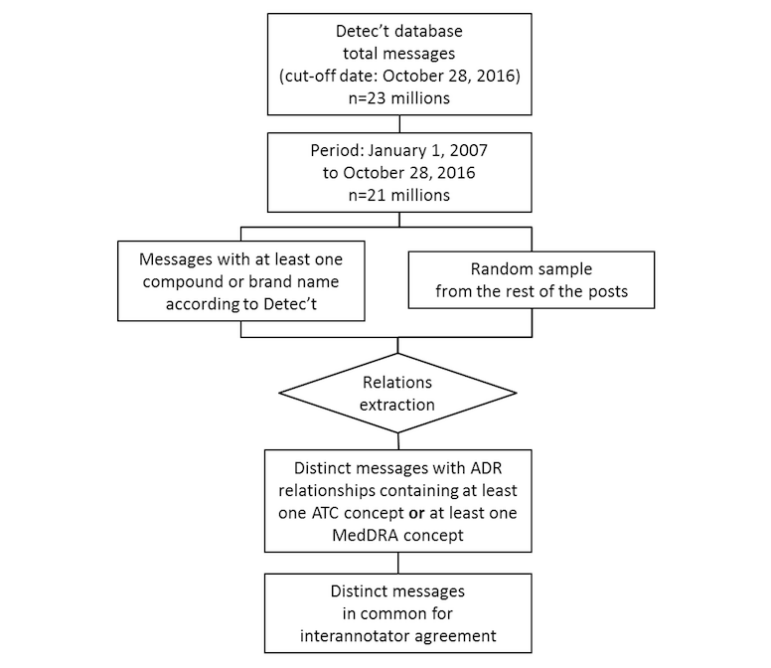
Phase 2 messages selection flowchart. ADR: adverse drug reaction; ATC: Anatomical Therapeutic Chemical (in ATC classification); MedDRA: Medical Dictionary for Regulatory Activities.

For phase 2, the selection of datasets is given in Figure 3.

On October 28, 2016, Kappa Santé Detec’t database contained about 23 millions of posts, and approximately 21 million posts were published after January 1, 2007. Messages about drugs, for which marketing authorization has been withdrawn before the cut-off date, were considered as some AEs might appear at long term or patients might discuss about old drugs and their safety.

The software developed by Kappa Santé for purpose of Web discussions collection offered the possibility of preidentifying the posts containing at least one pharmaceutical or molecule name. We considered the dataset of all posts containing at least one preidentified compound or brand name and combined it with a complementary set of posts randomly sampled from the remainder of the Kappa Santé Detec’t database.

The drug, AE, and ADR extraction modules developed for ADR-PRISM were executed on both sets of posts, and the output of the extraction module was considered as the evaluation dataset.

##### Drug Selection

The evaluation focused on 3 categories of drugs: (1) the most frequently extracted drugs in the corpus, (2) the most sold drugs in France, and (3) the drugs of interest of pharmacovigilance because of recent safety alerts. For the last 2 categories, the frequencies of the recognized named entities were also taken into account to cope with the necessary sample size.

The most sold drugs in 2013 in France are listed below, in their French commercial names, including the 10 most sold drugs with *mandatory* medical prescription for dispensation and the 10 most sold drugs with *optional* medical prescription for dispensation [22]:

- *Mandatory medical prescription for dispensation:* Levothyrox, Uvedose, Lamaline, Dafalgan codéiné, Méthadone, Crestor, Pivalone, Seresta, Emla patch, and Seroplex
- *Optional medical prescription for dispensation:* Doliprane, Dafalgan, Efferalgan, Kardégic, Spasfon, Gaviscon, Dexéryl, Météospasmyl, Biseptine, and Eludril.

The 2 experts additionally worked out a list of pairs of 1 drug concept and 1 AE concept corresponding to alerts that emerged in the last years (Table 1). This list of use cases has been further employed as a basis for the concept selection for evaluation. The concept selection in phase 1 (iterative improvement) [21] was based on the same consideration; therefore, those concepts on which the extraction tool performed well should be excluded from the selection for evaluation.

**Table 1:** List of drug and adverse event concepts selected as pharmacovigilance use cases.

Active drug ingredient: French names (date of marketing in cases where drug has been withdrawn)	Adverse events of interest	Corresponding Medical Dictionary for Regulatory Activities term	Media coverage or alert date
Gardasil	Autoimmune disease, complex regional pain syndrome, and postural orthostatic tachycardia syndrome	Autoimmune disorders (HLGT^a^) + complex regional pain syndrome (PT^b^) + postural orthostatic tachycardia syndrome (PT)	2013
Meningitec	Quality defect and any adverse event	All terms	2015
Methylphénidate: Ritaline, Concerta, Medikinet, Quasym, and Ritaline	Neurological and cardiac effects	Cardiac disorders (SOC^c^) + nervous system disorders (SOC)	2009 (recommendation)
Acétate de cyprotérone: Diane 35, Cypropharm (2010-2013), Elleacnelle (2010-2013), Evepar, Holgyme (2002-2015), Lumalia (2003-2013), Minerva, and Arielle (1997-2011)	Thromboembolic risk	Embolism and thrombosis (HLGT)	2012 (alert)
Fluoxétine: Prozac, Elindra (2001-2008), Fluctine (1998-2008), and Fontex (2001-2008)	All adverse events	All terms	Mostly 2013
Méthadone	Prolonged time from the start of the Q wave to the end of the T wave during electrocardiogram (approximates the time taken from when the cardiac ventricles start to contract to when they finish relaxing)	QT interval prolongation	2007
Sofosbuvir: Harvoni, Epclusa, and Sovaldi	Bradycardia	Bradycardia (PT)	2013
Codeine: Codenfan, Codoliporane, Migralgine, and Néocodion et Prontalgine	Respiratory disorders	Respiratory disorders (HLGT)	2012 re-evaluation
Hydroxyzine: atarax	Rhythm disorders	Cardiac arrhythmias (HLGT)	2014
Nicorandil: Adancor and Ikorel	Skin ulceration	Skin ulcer (HLT^d^)	2012
Midodrine: Gutron	Hypertension	Blood pressure increased (PT)	2013
Galantamine: Reminyl, Galanthen (2012-2015), and Galema (2011-2015)	Skin reaction	Skin and subcutaneous tissue disorders (SOC)	2014
Crizotinib: Xalkori	Heart failure	Heart failures HLGT	2014
Valproate de sodium: Depakine, Imaslav, and Micropakine	Teratogenic effects	Congenital, familial, and genetic disorders (SOC)	2012?
Isotrétinoine: Curacné, Acnetrait, Contracné, Procuta, and Roaccutane	Teratogenic effects + psychiatric disorders	Congenital, familial, and genetic disorders (SOC) and psychiatric disorders (SOC)	2002
Fingolimod: Gilenya	Leukoencephalopathy	Toxic leukoencephalopathy (PT)	2014
Aripiprazole: Abilify	Suicidal behavior	Suicidal and self-injurious behavior (HLGT)	2013 (words of warning: 2016)

^a^HLGT: high-level group term.

^b^PT: preferred term.

^c^SOC: system organ class.

^d^HLT: high-level term.

##### Adverse Event Concepts Selection

The AE concepts for evaluation corresponded to preferred term (PT) level in the MedDRA hierarchy, and we focused similarly on 2 categories: (1) the most frequent extracted PTs and (2) the PTs of interest of pharmacovigilance (Table 1), guided also by the frequencies of the recognized entities in the corpus to cope with the necessary sample size.

##### Adverse Drug Reaction Definition

We defined an ADR as any explicit and positive relationship between a drug and an AE where the drug (respectively the AE) belonged to the list of concepts previously selected.

##### Corpus for the Evaluation (Random Sample From Evaluation Dataset)

Finally, a random sample from the evaluation dataset (ie, among all the ADR extracted by the ADR information extraction module) constituted our corpus for evaluation.

### Gold Standard

#### Adverse Drug Reactions From Patient Reports in Social Media Manual Annotation Platform

We developed a Web application dedicated to manual annotations valuable as gold standard, for the project purposes in phase 2.

**Figure 4:**
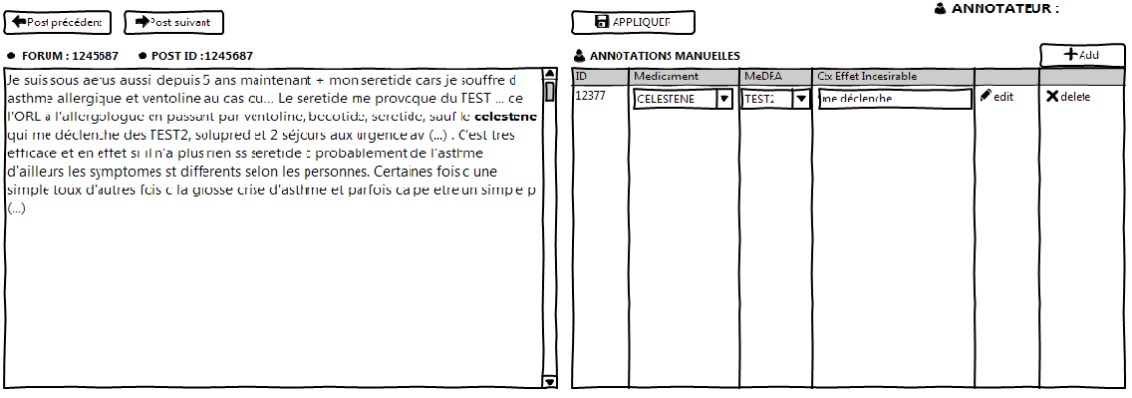
Interface of the manual annotation platform. ID: identifier; MedDRA: Medical Dictionary for Regulatory Activities.

This application was based on Java Servlets and JavaScript libraries and connected in Java Database Connectivity to the dataset of posts selected for the gold standard annotation. We used a self-completion mechanism to attach portions of message to *Racine Pharma* and MedDRA terms. We used drag-and-drop operations to fill in a table containing the manual review of the co-occurrences, where each line was dedicated to 1 co-occurrence. For each co-occurrence, the drug and the AE were dragged-and-dropped in the first and the second column, respectively. In the third column, the manual annotator was given a drop-down menu that presented 3 possibilities for defining the co-occurrence: explicit positive ADR, explicit negative ADR, or no ADR. We finally offered the possibility to export this table containing the manual annotations obtained via the application. The interface of this application is shown in Figure 4.

#### Gold Standard Construction

An annotator guideline has been established to standardize manual annotations. A total of 2 experts in medical terminologies have annotated all ADR relationships for each post in the evaluation corpus, and then, the manual annotation was considered as gold standard. Different from phase 1, here we expected that the annotators annotated all causal relationships between a drug and an AE, even if the drug and AE were not in the same sentence, which will allow us further evaluate the impact of the sentence boundary constraint of the extraction tool.

Each annotator annotated 55.1% (261/474) of the posts in the evaluation corpus; thus, a subset of 10.1% (48/474) of the posts was annotated by both of them. The interannotator agreement could be assessed on these double-annotated posts. In case of disagreements, the 2 annotators discussed to achieve a consensus. If a lot of disagreements had occurred, the annotators would have been asked to learn again the guideline and revise their annotations. All along the process, both annotators were double-blinded: first, from the ADRs identified by the ADR information extraction module, and second, from the other annotator’s annotations.

### Statistical Analyses

#### Primary Analysis

To assess the efficacy of the ADR information extraction module as compared with the gold standard, we will globally calculate the recall, precision, and f-measure.

**Figure 5:**
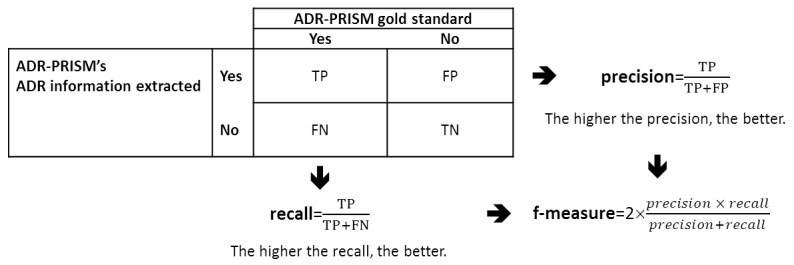
Recall, precision, and f-measure definitions. ADR: adverse drug reaction; ADR-PRISM: Adverse Drug Reaction from Patient Reports in Social Media; FN: false negative, manually annotated ADR by the gold standard that is not extracted by the ADR information extraction tool; FP: false positive, positive explicit relationship extracted by the ADR information extraction tool that is not manually annotated by gold standard; TN: true negative; TP: true positive.

In a post, if the ADR information extraction module identifies the same expression from *Racine Pharma* at the same position; *and* the same AE expression at PT level from MedDRA hierarchy at the same position; *and* the same type of relationship, as did the gold standard, then we will count the extracted *ADR* as a true positive (Figure 5).

#### F-Measure Gold Standard’s Evaluation

The interannotator agreement will be assessed by a hierarchical Kappa [23]. A hierarchical Kappa will enable to take into account the situation where the 2 annotators will disagree in either the level or the expression in ATC or MedDRA terminologies but will agree on higher levels than the annotated ones. Separate calculations for interannotator agreements by hierarchical Kappa on drug and AE expressions will be provided as well.

#### Secondary Analyses

We will complete these principal results with the following analyses.

We will take into account MedDRA and ATC granularities by reproducing precision, recall, and f-measure at each level of the hierarchies (system organ class [SOC], high-level group term [HLGT], high-level term [HLT], and preferred term [PT] of MedDRA and anatomical main group, therapeutic subgroup, pharmacological subgroup, and chemical subgroup of ATC).

We will provide a relaxed definition of true-positive ADRs combining the following 3 conditions: (1) the positions in the post of the extracted expressions for drug and AE both match the positions of the drug and AE expressions manually annotated, (2) the extracted and the manually annotated expressions for AE from MedDRA hierarchy are found in the same SOC levels, and (3) the classification of the identified relationship match the classification of the manually annotated relationship. We will calculate recall, precision, and f-measure with this definition for ADRs, drug, and AE identification separately.

We will provide a global estimation of the performance of ADR information extraction module by taking into account other types of messages. These messages are those without information on at least one ADR concept, that is, messages in which (1) ADR information is extracted but is not concerned by the drug- or AE-selected concepts, (2) no ADR information is extracted but only co-occurrences of information about 1 drug and 1 AE, (3) only AE information is extracted, (4) only drug information is extracted, and (5) neither drug nor AE information is extracted. After having sampled each type of message described above, we will then calculate global recall or precision or f-measure on all types of messages by marginal calibration.

All analyses will be performed in *R* software [24].

### Sample Size Calculation

Recall can be estimated from a random sample of ADR concepts annotated by the gold standard, whereas precision can be estimated from a random sample of ADR concepts extracted by the ADR information extraction module. However, creating the gold standard on the whole evaluation dataset before sampling in it for the recall was an overwhelming task. At the same time, a vast majority of messages did not contain any information on the drug concept. Thus, we expected a very low proportion of ADR concepts identified by the ADR information extraction module in the evaluation dataset. Therefore, the recall estimated from a random sample of ADR concepts annotated by the gold standard was mathematically approximated by the recall estimated on ADR concepts sampled for the precision.

The sample size required for different expected precision or recall is provided in Table 2. By hypothesizing a global recall of 0.5, we needed at least 384 identified ADR concepts to obtain a 95% CI, having a total width of 0.1. The final determination of sample size was based on the previously estimated precision and recall.

**Table 2:** Sample size required according to the expected precision and recall and total width of confidence intervals (CIs).

Range of CI	Expected precision or recall values								
	0.1	0.2	0.3	0.4	0.5	0.6	0.7	0.8	0.9
90% CI	35	61	81	92	96	92	81	61	35
95% CI	138	246	323	369	384	369	323	246	138

### Differences Between Phase 1 and Phase 2

The main differences between phase 1 and phase 2 are threefold (Table 3). First, in phase 1, the annotations targeted all drugs and AEs even if not involved in an ADR relation, whereas in phase 2, annotation focuses on the ADR relations. Second, in phase 2 (and only in phase 2) a gold standard based on blind manual annotation by 2 experts in medical terminologies was established and the interannotator agreement was measured. In contrast, the manual annotations of phase 1 were not blind, and most of the work consisted of validating the automatic annotations; thus, the interannotator agreement was not addressed. Finally, in phase 2, additional parameters such as the granularity and the segmentation of the messages (sentence boundaries) will be analyzed. The precision, recall, and f-measure will be calculated with a gold standard at the message level and might be compared with precisions, recalls and f-measures calculated at the sentence level and for all MedDRA and ATC granularities.

**Table 3:** Common features and differences between phase 1 and phase 2.

Issues	Phase 1	Phase 2
Evaluation focus	Drugs and adverse events entity recognition	Adverse drug reaction relationships recognition
Gold standard (manual annotations)	Not blind	Blind
Gold standard annotators	Pharmacovigilance experts	Experts in medical terminologies
Interannotator agreement	No	Yes
Granularity issue	No	Yes
Sentence boundary issue	No	Yes

## Results

At the time of publishing this evaluation protocol, several steps of this project would have been completed. The corpus for evaluation has been constituted, and the NLP tools have identified and extracted the information about ADR inside this corpus. We selected the ADR concepts and constituted the samples of the entities necessary to set up the gold standard. The 2 annotators completed the annotations process.

Data analyses for assessing the interannotator agreement and the performance of the ADR information extraction module as compared with the gold standard are ongoing, and the study results are expected internally before the end of 2018.

## Discussion

### Summary

With the objective of using messages on health forums as a new source for drug safety, the systems developed to mine the messages must follow strict evaluation rules. This is even more important as these systems might be used to support decision making. The protocol presented in this paper has been designed to evaluate the ADR information extraction Skill Cartridge developed in ADR-PRISM in a pharmacovigilance perspective.

### Study Strengths

The protocol presented in this paper is an attempt to apply clinical research–level guidelines [14] to the assessment of such systems.

First, we paid particular attention to the establishment and the validation of the gold standard. The gold standard was established by 2 trained specialists of medical terminologies who annotated selected messages. Manual annotation was performed in a *double-blind* manner, namely, from both the other annotator and the ADR identified by the ADR information extraction module. The annotated corpus, therefore, constituted a valuable gold standard. On the basis of the study by Sarker et al [4] (see Multimedia Appendix 1), we could find 13 studies where a gold standard, that is, manual annotations used for evaluation, was implemented [25-37]. Conversely, in 8 studies, there was no gold standard [38-45], and the extracted ADRs were compared with already known AEs from FAERS [39-41,43,44] or drug label declared to FDA [38,45] or even AE described in websites [42]. Moreover, in the ADR-PRISM protocol, a common subset of randomly selected messages was annotated in a blind manner by the 2 experts; interannotator agreement evaluation will also ensure ourselves about the quality of this gold standard. In most of studies, the authors gauged the interannotators agreement [25,27,30-32,34,35]. However, it is not systematic [26,28,29,33,36,37].

Second, by calculating a sample size of messages collected from social media, to assess recall, precision, and f-measure, we guaranteed the statistical power to place reliance on our study results.

The chosen terminologies are another crucial aspect of this work. On the one hand, the *Racine Pharma* thesaurus, with 5164 entries, exhaustively covers a large range of drug names and active ingredients. On the other hand, the MedDRA hierarchy is used daily by drug safety experts and considered expressive for this task. By using these terminologies, we expect to increase the sensitivity of the ADR-PRISM Skill Cartridge for pharmacovigilance. Our choice to map *Racine Pharma* to ATC was guided by 2 aspects. First, ATC like MedDRA has a hierarchical structure. Thus, we will be able to evaluate the ADR information extraction tool based on different hierarchical levels of these terminologies. Second, ATC and MedDRA are widely used and internationally agreed reference terminologies. Hence, we expect to provide strong and reproducible results.

The ADR information extraction module was not only based on drug and AE information identification but also on rules and regular expressions. As such, we expect to discard noninformative sentences, addressing general information about the drug or drug indications. We would also be able to identify unexpected *positive* effects, as for example, headaches that would be reduced by a drug without indication for the treatment of this kind of pain. Only few studies have been able to take this aspect into account [27,29,30,32,34,46].

### Study Limitations

Despite its positive aspects, the study exhibits several limitations.

The mapping between the terms listed in the drug thesaurus *Racine Pharma* and the terms referenced in the chemical substance in the ATC hierarchy was incomplete. Among the 5164 terms in *Racine Pharma*, 852 (16.5%, 852/5164) could not be mapped to ATC, for example, some phytotherapies (St John’s wort herbal tea, Silver birch juice, extract of licorice root, arum triphyllum compound, arnica, etc). This could have a negative impact on the recall scores calculated according to the ATC levels.

In social media’ posts, slangs and colloquial languages are frequent; likewise, syntactic rules are approximate. We chose to use fuzzy-matching and thesaurus enrichment to take into account this bias. However, this approach is inherently nonexhaustive with negative impact on the recall of the ADR information extraction module.

Regarding the gold standard, 2 experts in medical terminologies trained in drug safety performed manual annotations. However, contrary to the phase 1 manual review, none of them could be considered as a pharmacovigilance expert.

The medical informatics community needs shared open corpora to evaluate their methods and tools. Recent efforts have led to making several datasets more accessible and the evaluation of the methods more standardized, for example, the Multiparameter Intelligent Monitoring in Intensive Care corpus [47] and the Informatics for Integrating Biology and the Bedside challenges [48]. Drug safety could benefit from open-access validation datasets made available for research purposes [25,35]. However, several obstacles remain. For example, the ADR-PRISM consortium got approval to reuse posts for research purposes, but the approval is restricted to the project.

### Conclusions

The objective of this study is to present the scientific approach developed in the first stage of the ADR-PRISM project. At this stage, our principal objective was to evaluate the performance of the ADR information extraction Skill Cartridge against a gold standard constituted by human annotations. To address this question, our goal was to adapt the principles adopted in clinical research to assess sound and trustworthy measurements of precision, recall, and f-measure.

With the statistical theory, we calculated a sample size. This guaranteed enough ADR information and sufficient narrowness of the confidence interval, to scientifically conclude on our principal objective. We also avoided unnecessarily large extractions for which the process of manual annotations is nothing but both wasteful and time-consuming.

## Appendix

Multimedia Appendix 1 Designs of articles related to ADR information extraction on social media (replicated from Sarker et al).

## Abbreviations

ADR: adverse drug reaction
ADR-PRISM: Adverse Drug Reaction from Patient Reports in Social Media
AE: adverse event
ATC: Anatomical Therapeutic Chemical (in ATC classification)
FAERS: Food and Drug Administration’ adverse event reporting system
FDA: Food and Drug Administration
HLGT: high-level group term
HLT: high-level term
MedDRA: Medical Dictionary for Regulatory Activities
NLP: natural language processing
PT: preferred term
SIBM-CISMeF: Biomedicine informatics service catalogue and index of French language medical websites (French Service d’Informatique Biomédicale-Catalogue et Index des Sites Médicaux de langue Française)
SOC: system organ class
