# Supplementary material for: The Adverse Drug Reactions From Patient Reports in Social Media Project: Protocol for an Evaluation Against a Gold Standard
Source: JMIR Res Protoc. 2019 May 7;8(5):e11448. doi: 10.2196/11448 (PMC6528435; doi:10.2196/11448)
Supplement: Multimedia Appendix 1 [file resprot_v8i5e11448_app1.pdf]

Supplementary file 1 Designs of articles related to ADR information extraction on social media (replicated from Sarker et al, reference 4 in the main manuscript)

Author(s) year	Social media	Corpus size & number of drugs	Compa-rator [a]	Gold standard (GS) features			
				Corpus size[a]	Randomly sampled[a]	Sample size calcula-tion[a]	GS perfor-mance evaluation [a]
Benton 2011 [42]	11 websites (breastcancer.org, komen.org, csn.cancer.org, bcsupport.org, healthboards.com, cancercompass.com, webmd.com, dailystrength.org, dailystrength.org, revolutionhealth.com, ehealthforum.com, oprah.com)	1,100 messages; 4 breast cancer drugs	Drug label	-	-	-	-
Bian 2012 [27]	Twitter	2,000,000,000 tweets; investigational drugs used in cancer treatment clinical trials	FAERS (FDA)	-	-	-	-
Chee 2011 [43]	Health and Wellness Yahoo! groups	12,519,807 messages; 4 drugs withdrawn from the market	FDA watchlist (drug that have an active FDA safety alert posted)	-	-	-	-
Freifeld 2014 [28]	Twitter	61,401 (or 61,402) tweets incl 4,401 *proto-AEs (posts with 'resemblance to AEs') vs 57,000 no proto-AEs; 23 drugs	AEs in FAERS at SOC level	-	-	-	-
Ginn 2014 [29]	Twitter	71,571 tweets; 74 drugs	2 annotators with medical or	10,822 tweets	yes	Maximum 300-500 tweets per	P,R, Fscore vs 1st annotator + Cohen's

Author(s) year	Social media	Corpus size & number of drugs	Compa-rator [a]	Gold standard (GS) features			
				Corpus size[a]	Randomly sampled[a]	Sample size calcula-tion[a]	GS perfor-mance evaluation [a]
			biological science background; Weekly meetings for confused instances. For this paper, the 1st annotator = Gold Standard			drugs	Kappa
Hadzi-Puric 2012 [30]	8 parenting forum	1,290 posts; 9 drugs	2 annotators experienced in pharmacy and research, clinical research and pediatric care practice) + literature	990	NR	NR	NR
Jiang and Zheng 2013 [44]	Twitter	6,829 tweets; 5 drugs	NLM MedLinePlus Drug Information Site ; PatientsLikeMe	-	-	-	-
Leaman 2010 [31]	DailyStrength	6,890 comments; 6 drugs, only 4 with annotations	2 annotators experienced in biology, nursing, clinical research, drug surveillance	3,600 incl. 450 (system development) + 150 (validation)	yes	NR	Cohen's kappa (w/o span), IAA (w/ span)
Liu et Chen 2013 [32]	American Diabetes Association (community.diabetes.org)	185,874 posts; 1,348,364 sentences; - drugs	Manual annotation	200 relation instances	NR	NR	NR
Nikfarjam	DailyStrength	6,890	2 experts	3,600	yes	NR	NR

Author(s) year	Social media	Corpus size & number of drugs	Compa-rator [a]	Gold standard (GS) features			
				Corpus size[a]	Randomly sampled[a]	Sample size calcula-tion[a]	GS perfor-mance evaluation [a]
and Gonzales 2011 [33]		comments; 4 drugs	annotators	annotated comments; incl. 1,200 comments (evaluation)			
Nikfarjam and Sarker 2015 [34]	DailyStrength, Twitter	DailyStrength: 6,279 comments, Twitter: 1,784 tweets + Unsupervised learning on DailyStrength 313,833 comments, Twitter: 397,729 tweets; 81 drugs	2 experts annotators supervised by an expert pharmacologist	6,279 DailyStrength incl. 4720 (training)+1559(validation); 1,784 tweets incl. 1340 (training)+444 (validation), with complete IAA only	NR	NR	Cohen's kappa (approximate matching)
O'Connor 2014 [35]	Twitter	1,873 tweets; 54 drugs	2 expert annotators	10,822 (classification 'hasADR' vs 'noADR'); 1,873 (automatic ADR information extraction = 1,008 'hasADR' + ca 1,000 random selection of 'noADR')	yes	Maximum 300-500 tweets per drugs	IAA (span, concept ID, binary classification) + kappa (binary classification)
Patki 2014 [36]	Dailystrength	20,486 comments 10,399 normal drugs, and 7,327 drugs with FDA-issued blackbox warning; and 2,760 withdrawn drugs10,617 for Machine Learning; 38 chronic disease and conditions drugs AND	2 domain experts under the guidance of a pharmacology expert	10,617 user comments	NR	NR	Cohen's kappa

Author(s) year	Social media	Corpus size & number of drugs	Compa-rator [a]	Gold standard (GS) features			
				Corpus size[a]	Randomly sampled[a]	Sample size calcula-tion[a]	GS perfor-mance evaluation [a]
		high prevalence of use (20 normal + 18 with FDA blackbox warning)					
Sampathkumar 2014 [37]	medications.com; steadyhealth.com	Medications.com: 8,065 posts SteadyHealth.com: 29,981 posts; 760 common drug names	Manual review	2,000 messges (medications.com) incl. 500 msg automatically annotated positive + 1500 msg automatically annotated negative	NR	NR	NR
Sarker and Gonzales 2015 [38]	Twitter, DailyStrength	Twitter: 10,617 tweets; 74 drugs DailyStrength: Over 25,000 comments; 56 drugs	2 manual annotators under guidance of a 1 pharmacology expert; disagreement resolved by pharmacology expert	10,822 instances (Twitter) incl. 1,082 tweets (10% of corpus) for IAA; 10,617 instances(DailyStrength), incl. 10% of corpus) for IAA	yes	10% of corpus	Cohen's kappa
Segura-Bedmar 2014 [39]	ForumClinic	400 comments; 187 drugs	2 annotators with expertise in pharmacovigilance; disagreement resolved by a third annotator	400 comments	yes	NR	Fscore
Yang and Yang and Jiang and Zhang 2012 [46]	MedHelp	6,244 threads; 10 drugs	FDA watchlist	-	-	-	-
Yang and Wang 2013 [40]	Yahoo ! groups (ProzacAwareness, SSRIsex)	6,400 posts; 2 drugs	3 independent medical domain	6,400 posts	no (entire GS corpus)	200 most frequent ADRs descripti	NR

Author(s) year	Social media	Corpus size & number of drugs	Compa-rator [a]	Gold standard (GS) features			
				Corpus size[a]	Randomly sampled[a]	Sample size calcula-tion[a]	GS perfor-mance evaluation [a]
			experts			on keyworker; then 900 threads from each forum which contain the most mentions of these ADRS	
Yates and Goharian 2013 [41]	askpatient.com; drugs.com; drugratingz.com	2,500 user reviews; 5 breast cancer drugs	Manual annotation	125 (training) + 125 (validation)	NR	10% of the corpus	NR
Yeleswarapu 2014 [47]	PatientsLikeMe, DayliStrength MediGuard	13,500 comments ; 12 drugs (from Wang JAMIA 2009 and Leaman 2010)	FDA Label information	-	-	-	-
Yang and Yang and Jiang 2014 [45]	MedHelp	16,339 threads; 20 drugs extracted with more than 500 threads	FDA SRS (MedWatch); 5 ADR alerted by FDA chosen on the principle that the drugs should share as many ADRs as possible	-	-	-	-

[a] additional columns to [1]
IAA inter annotator agreement
NA not applicable
NR not reported
P precision
R recall

Reference:

Sarker A, Ginn R, Nikfarjam A, O'Connor K, Smith K, Jayaraman S, et al. Utilizing social media data for pharmacovigilance: a review. J Biomed Inform 2015 Apr; 54: 202-212
